# APOE ε4 Gene Carriers Demonstrate Reduced Retinal Capillary Densities in Asymptomatic Older Adults

**DOI:** 10.3390/jcm12175649

**Published:** 2023-08-30

**Authors:** Ziyi Zhang, William Robert Kwapong, Le Cao, Zijuan Feng, Bo Wu, Junfeng Liu, Shuting Zhang

**Affiliations:** Department of Neurology, West China Hospital, Sichuan University, Chengdu 610041, China

**Keywords:** APOE genotype, cognition, optical coherence tomography angiography, retinal microvasculature, Alzheimer’s disease

## Abstract

Early identification of Apolipoprotein E (APOE)-related microvascular pathology will help to study the microangiopathic contribution to Alzheimer’s disease and provide a therapeutic target for early intervention. To evaluate the differences in retinal microvasculature parameters between APOE ε4 carriers and non-carriers, asymptomatic older adults aged ≥ 55 years underwent APOE ε4 genotype analysis, neuropsychological examination, and optical coherence tomography angiography (OCTA) imaging. One hundred sixty-three older adults were included in the data analysis. Participants were also defined as cognitively impaired (CI) and non-cognitively impaired (NCI) according to their MoCA scores and educational years. APOE ε4 carriers demonstrated reduced SVC (*p* = 0.023) compared to APOE ε4 non-carriers. Compared to NCI, CI participants showed reduced SVC density (*p* = 0.006). In the NCI group, no significant differences (*p* > 0.05) were observed in the microvascular densities between APOE ε4 carriers and non-carriers. In the CI group, APOE ε4 carriers displayed reduced microvascular densities compared to non-carriers (SVC, *p* = 0.006; DVC, *p* = 0.048). We showed that CI and APOE ε4 affect retinal microvasculature in older adults. Quantitative measures of the retinal microvasculature could serve as surrogates for brain microcirculation, providing an opportunity to study microvascular contributions to AD.

## 1. Introduction

Alzheimer’s disease (AD), the most prevalent cause of dementia, is strongly genetically linked to the APOEε4 genotype, the most potent risk factor for sporadic Alzheimer’s disease [1,2]. The precise role of the APOE gene in AD pathogenesis remains the subject of ongoing investigation. Nonetheless, several studies [3,4,5] have suggested a potential connection between the APOE gene and vascular health, indicating the possible involvement of vascular pathways in the development of AD.

Cerebral imaging studies [4,6,7,8] using magnetic resonance imaging (MRI) have shown that APOE ε4 carriers display significant cerebral microstructural and vascular changes compared to APOE ε4 non-carriers. Additionally, the study suggested that APOE ε4-mediated neurodegeneration is related to vascular pathology [9]. These investigations imply that APOE ε4-related microvascular impairment might play a part in AD’s pathological landscape. However, the exact mechanisms by which these APOE ε4-mediated microvascular alterations contribute to AD pathology are not entirely clear. In recent research [10], it was shown that mice carrying human APOE ε4 alleles exhibit capillary pathology, a compromised blood–brain barrier (BBB), and impaired function of the neurovascular unit (NVU). MRI has undoubtedly provided valuable indirect insights into APOE ε4-related cerebral pathology, such as white matter hyperintensity, microbleed, and enlarged perivascular space. However, MRI cannot directly realize the visualization of cerebral microvasculature, and it has limited availability due to high costs, inaccessibility, and the need for a professional post-processing system. Those highlight the need for more accessible and cost-effective biomarkers. Identifying such markers could facilitate the early detection of cerebral microvasculature changes and improve our understanding of APOE’s role in AD pathophysiology, develop biomarkers for early diagnosis, and provide therapeutic targets for early intervention.

The retina, a neurosensory tissue in the eye, and the brain share similar characteristics, such as precise neuronal cell layers, microvasculature, and embryologic origin [11]. The potential for early detection of disease-related biomarkers in Alzheimer’s disease through retinal imaging is promising due to its direct visual connection to the retina and shared neurobiology with the brain. Previous studies [12,13] using fundus photographs have shown that quantitative changes in retinal vessel width (narrower arteriolar caliber and wider venules) and reduced fractal dimension are associated with AD patients. Furthermore, some reports [12,13] have demonstrated that these retinal microvascular changes are linked with cognitive performance in AD patients. Recent reports [14,15] utilizing fundus photography have revealed the noteworthy role of APOE gene polymorphism in the development of retinal microvascular changes among older adults.

Optical coherence tomography angiography, an imaging technique, offers a non-invasive way to depict the retinal microvasculature with exceptional clarity [16,17]. Unlike earlier retinal imaging methods like fundus angiography [17], this technology produces detailed three-dimensional (3D) images of the retinal structure and microvasculature across various layers. OCTA has been extensively applied in neurological diseases such as AD [12,13,18] and Parkinson’s disease [19,20]; these reports showed patients with these neurological disorders had reduced retinal microvascular densities and sparser retinal microvasculature. Taken together, these reports suggest that OCTA has the potential to be used as a tool to detect and monitor microvascular changes in these neurological diseases. Previous studies investigated the effect of the APOE gene on retinal microvasculature in symptomatic AD and dementia; however, little is known about the effect of APOE on retinal microvasculature in the preclinical disease stage.

As part of the current study, we utilized OCTA’s established sensitivity to detect subclinical capillary pathology to investigate APOEε4-associated microvascular disease in older asymptomatic adults.

## 2. Materials and Methods

### 2.1. Study Design

This study is part of an ongoing study assessing the connection between retinal biomarkers and neurodegenerative disease in older adults at the Department of Neurology, West China Hospital of Sichuan University. Inclusion in our study required participants to be ≥55 years of age, without complaints of memory decline, have intact visual function, speak Chinese Mandarin, and cooperate with retinal imaging. Eligible participants underwent neuropsychological evaluation, APOE gene analysis, blood investigations, MRI scans, and a thorough medical history review, including history of ophthalmic disease, cardiovascular risk factors such as smoking and drinking status, previous stroke, hypertension, and diabetes mellitus, and current prescribed medications, was conducted. Details of all participants were reviewed and discussed among neurologists, neuro-ophthalmologists, and clinical research fellows. Exclusion criteria from the study were as follows: individuals with a history of psychiatric or neurological disorders (such as dementia, Parkinson’s disease, stroke, traumatic brain injury, brain surgery, drug addiction, depression, schizophrenia, and so on), any significant systemic disease (heart failure, renal insufficiency, cancer, etc.), and current use of any medications known to affected cognition. Additionally, visual acuity ≤0.8 (LogMAR), uncontrolled hypertension, and uncontrolled diabetes mellitus were also employed as exclusion criteria. The protocol was approved by the Ethics Committee of West China Hospital of Sichuan University (2020-104). Written informed consent was obtained before data collection.

### 2.2. Neuropsychological Examination

Participants underwent the Montreal Cognitive Assessment (MoCA) and mini-mental state examination (MMSE). Participants were defined as cognitively impaired (CI) and non-cognitively impaired (NCI) by their MoCA scores and educational years, as previously reported [21,22]. The cut-offs are shown in Table S4.

### 2.3. Apolipoprotein E Gene Analysis

Blood samples were procured from the peripheral blood of individuals and examined at the West China Hospital of Sichuan University. Subsequently, the samples underwent amplification using the polymerase chain reaction (PCR) method through the utilization of an ABI 7500 FAST instrument from Applied Biosystems, Thermo Fisher Scientific, located in Waltham, MA, USA [23]. For the determination of APOE haplotypes, an APOE haplotype determination kit from Memorigen Biotech in Xiamen, China, was employed. This kit operated on the basis of fluorescent PCR technology and employed three pairs of detection reagents designed to selectively identify specific single nucleotide polymorphisms (RS429358 and RS7412) associated with APOE gene types 2, 3, and 4. This approach facilitated the identification and amplification of the genetic samples. In instances where the genetic sample’s APOE allele corresponded with the amplification system, a PCR amplification reaction was initiated within the system. The exonuclease activity located at the 5′ to 3′ terminus of DNA polymerase was utilized to enzymatically degrade DNA molecular probes labeled with fluorescence. Following this enzymatic degradation, the probe became responsive to a fluorescence signal upon stimulation, which was then detectable by the monitoring system. The categorization of an individual’s APOE ε4 carrier status as either positive (ε2/ε4, ε3/ε4, ε4/ε4) or negative (ε2/ε2, ε2/ε3, ε3/ε3) was established based on the obtained genetic analysis outcomes.

### 2.4. Ophthalmological Examination

#### 2.4.1. Visual Acuity (VA) Examination

Participants underwent visual acuity (VA) examination under light using the Snellen chart. VA for each eye was later converted to a logarithm of the minimum angle of resolution (LogMAR).

#### 2.4.2. Swept Source Optical Coherence Tomography Angiography (SS-OCTA) Imaging

The SS-OCTA tool (VG200; SVision Imaging, Henan, China) contained a central wavelength of 1050 nm and a scan rate of 200,000 A-scan per second. The tool was set with an eye-tracking function based on an integrated confocal scanning laser ophthalmoscope to remove eye-motion artifacts. The specifications of the OCTA tool are well described in our previous reports [24,25].

**Figure 1:**
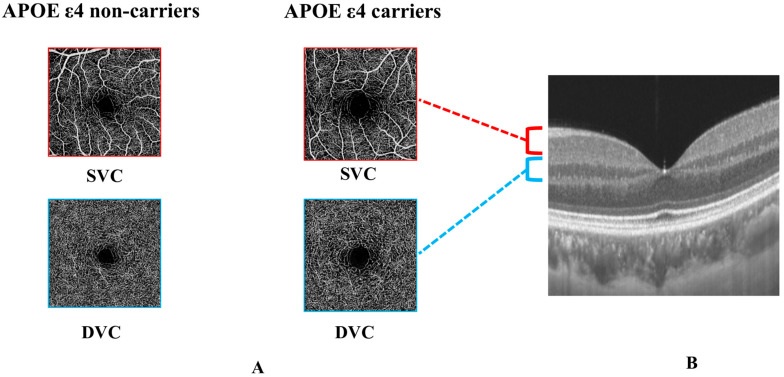
Comparison of OCTA parameters between APOE ε4 non-carriers and APOE ε4 carriers and segmentation of retinal microvasculature. (**A**) shows the comparison of SVC and DVC density between APOE ε4 non-carriers and APOE ε4 carriers. (**B**) shows the segmentation between the SVC and the DVC was set in the inner two-thirds and outer one-third interface of the ganglion cell layer and inner plexiform layer (GCIPL).

En face angiograms of the superficial vascular plexus (SVC) and deep vascular plexus (DVC) were obtained by the OCTA tool in a 3 mm × 3 mm area centered on the fovea. The inner retina, containing the SVC and DVC, extended from 5 µm above the inner limiting membrane (ILM) to 25 µm below the lower border of the inner nuclear layer (INL). The segmentation (Figure 1) between the SVC and the DVC was set in the inner two-thirds and outer one-third interface of the ganglion cell layer and inner plexiform layer (GCIPL). Microvascular densities of each plexus were measured by the OCTA tool. OCTA data displayed in our study followed the OSCAR-IB quality criteria [26] and APOSTEL recommendation [27]. The exclusion criteria of our participants were as follows: confounding ocular disorders such as diabetic retinopathy, hypertensive retinopathy, severe cataracts, age-related macular degeneration (AMD), glaucoma, and history of vitreoretinal or optic nerve disease. If a participant presented with any of these disorders in one eye, the other eye was used; if both eyes had the aforementioned disorders, the participant was excluded from the study.

### 2.5. Statistics Analysis

Continuous variables were described by mean ± standard deviation (SD), and categorical variables were presented as frequency and percentages. The *t*-test was used for continuous variables, and the Chi-square test was used for categorical variables when comparing the demographic and clinical characteristic differences between the two groups. Generalized estimating equations (GEE) with multiple linear regression were used to investigate the retinal microvascular differences between APOE ε4 carriers vs. APOE ε4 non-carriers while adjusting for age, gender, education, vascular risk factors (diabetes, hypertension, hyperlipidemia, and smoking), and intereye dependencies, and CI vs. NCI while adjusting for age, gender, education, LogMAR, vascular risk factors (hypertension, diabetes mellitus, hyperlipidemia, and smoking), and intereye dependencies. GEE was conducted for subgroup analysis that investigated the retinal microvascular differences between APOE ε4 carriers vs. APOE ε4 non-carriers within the subgroups of individuals with CI and NCI while considering potential confounding factors as covariates. ANOVA analysis was used to perform interaction analysis to examine whether there was an interaction between APOE genotype and cognition status, and the results were presented in Supplementary Figure S1. To investigate the relative importance of APOE genotype and cognition to retinal microvasculature, we also built a multivariate linear model to examine their relative contribution. Finally, permutation tests [28] were used as sensitivity analysis for all parameter comparisons because of the sample size difference between the APOE ε4 carrier group and non-carrier group. The detailed method and results of this permutation test are shown in the Supplementary Materials (Supplementary Tables S2 and S3). *p*-values < 0.05 were considered statistically significant. Data analysis and plotting were performed in R version 4.0.3.

## 3. Results

**Figure 2:**
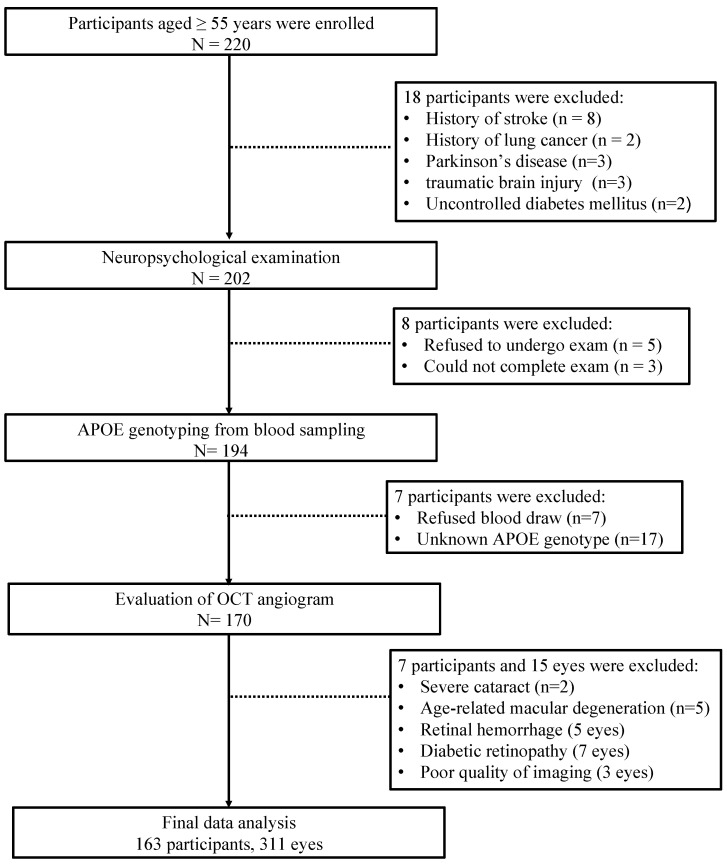
Flow chart illustrating the process of inclusion and exclusion of our study participants.

Figure 2 shows the flow diagram of our final participants. In total, 163 participants (62.58% females) and 311 eyes were included in our final analysis. Of the 163 participants, 35 participants were APOE ε4 carriers, and 128 participants were APOE ε4 non-carriers. Of note, 89 participants were grouped as NCI, and 74 were grouped as CI.

### 3.1. Baseline Analysis

No significant differences were observed in the age, gender, education, cognitive assessment, visual acuity, and vascular risk parameters between APOE ε4 carriers and APOE ε4 non-carriers (Table 1). Supplementary Table S1 shows no significant differences were seen in the demographics and clinical information of NCI and CI.

### 3.2. Retinal Vasculature Analysis of APOE ε4 Carriers vs. APOE ε4 Non-Carriers

**Figure 3:**
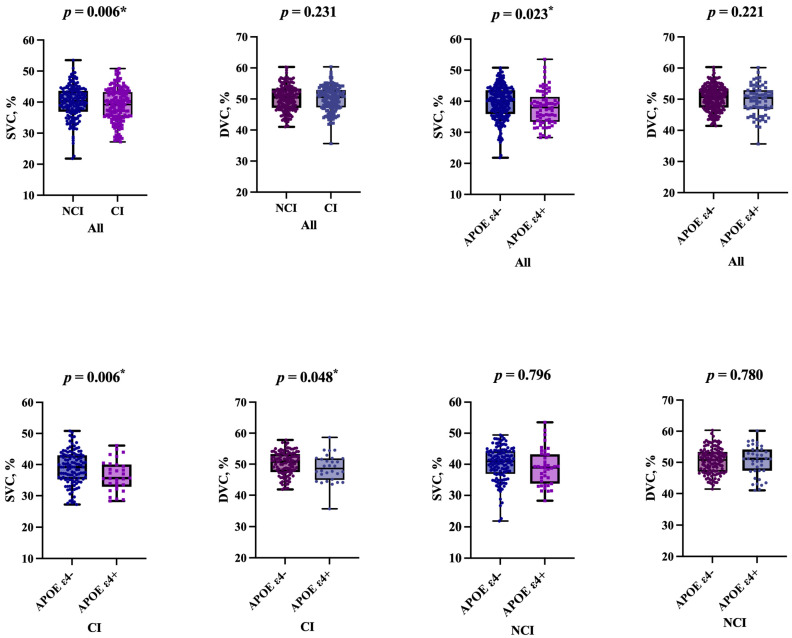
Comparison of OCTA parameters between APOE ε4 carriers vs. APOE ε4 non−carriers and CI vs. NCI. *: *p* value < 0.05.

Table 2 and Figure 3 show the results of OCTA parameter comparisons between APOE ε4 carriers and APOE ε4 non-carriers. APOE ε4 carriers showed reduced SVC density (*p* = 0.023) compared to APOE ε4 non-carriers in all participants when considering age, gender, education, diabetes, hypertension, hyperlipidemia, and smoking as covariates. However, there was no significant difference in the DVC density between the two groups (*p* = 0.221).

### 3.3. Retinal Vasculature Analysis of CI vs. NCI

Results of the OCTA parameter comparison of CI and NCI are presented in Table 2 and Figure 3. Compared to NCI, CI participants showed reduced SVC density (*p* = 0.006) in all participants after adjusting for age, gender, education, LogMAR, vascular risk factors (hypertension, diabetes mellitus, hyperlipidemia, and smoking), and intereye dependencies. No significant difference was observed in the DVC density when both groups were compared (*p* = 0.231).

### 3.4. Subgroup Analysis

Table 3 and Figure 3 provide the results of the subgroup analysis concerning OCTA parameters specifically related to APOE ε4 carriers. Following adjustments for age, gender, education, vascular risk factors (including hypertension, diabetes mellitus, hyperlipidemia, and smoking), as well as accounting for intereye dependencies, the outcomes of the subgroup analysis indicated the following: in the NCI group, no significant differences (*p* > 0.05) in microvascular densities were observed between APOE ε4 carriers and APOE ε4 non-carriers; conversely, within the CI group, APOE ε4 carriers exhibited notably reduced densities in SVC (*p* = 0.006) and DVC (*p* = 0.048) when compared to non-carriers.

### 3.5. Interaction Analysis

Figure S1 shows the results of interaction analysis, and there is no significant interaction between cognition and APOE genotype on SVC (*p* = 0.333).

### 3.6. Relative Importance Analysis

Table 4 illustrates the varying impacts of APOE genotype and cognitive factors on retinal vasculature. Notably, our analysis revealed that, when accounting for additional covariates such as age, gender, education, hypertension, diabetes mellitus, hyperlipidemia, and smoking, the influence of APOE genotype on the microvascular density of SVC was more pronounced compared to cognition.

## 4. Discussion

Despite extensive research on retinal microvasculature in AD patients and preclinical conditions, little is known about how APOE genetic factors affect the retinal microvasculature [13]. In our observational study, we demonstrated that APOE ε4 carriers reduced SVC density compared to non-carriers in older adults. We also showed CI participants had reduced SVC density compared to NCI. Importantly, in CI participants, APOE ε4 carriers demonstrated reduced microvascular densities compared to APOE ε4 non-carriers.

Using the SS-OCTA, our study provides further insights into the pathogenesis of cognitive impairment in older adults. Although cerebral microvascular impairment has been suggested to contribute to cognitive impairment, data to support such hypotheses are few [29]. Pathological studies [30,31] have shown AD is associated with a variety of structural and physiological changes in the cerebral microvasculature, including arteriolar narrowing, capillary damage, endothelial dysfunction, and blood–brain barrier damage. These structural microvascular changes may lead to abnormal microvascular flow patterns and neurodegeneration, eventually resulting in plaques and neurofibrillary tangles characteristic of AD and/or dementia. Retinal microvascular changes (specifically the superficial vessels) are suggested to reflect the cerebral microvasculature [32]. Using the retinal microvasculature as a proxy to the cerebral microvasculature, we showed CI participants had reduced SVC density compared to NCI. Reduced microvascular density in the SVC is thought to be indicative of a disturbed blood–retina barrier, disturbed blood flow, vessel wall dysfunction, and tissue hypoxia [12]. Thus, our study supports the hypothesis that microvascular impairment may play an important role in the development of cognitive impairment. Furthermore, these findings offer clues to the specific pathophysiological processes that occur in the cerebral microvasculature of older adults with cognitive impairment. Prior reports [12,13] showed individuals with cognitive impairment had wider venules and narrow arterioles. Of note, the retinal arterioles and venules are found in the SVC [33], suggesting reduced microvascular density in the SVC may be due to the structural microvascular changes associated with cognitive impairment. Taken together, we suggest that reduced SVC density may be an important pathological feature in the development of cognitive impairment in older adults.

The APOE ε4 genotype is the strongest common genetic risk factor for sporadic AD. Previous model reports demonstrated that APOE ε4 affects microvascular density and neurovascular regulation [5,34]. It is suggested that APOE ε4 carriers are associated with retinal vascular pathology independent of diabetes and hypertension [35]; individuals with APOE ε4 showed blot hemorrhages, indicative of blood–retina barrier damage [36]. Using the OCTA, a previous report [37] showed lower retinal capillary densities in cognitively normal APOE ε4 carriers when compared to non-carriers. In our current study, asymptomatic older adults with APOE ε4 carriers demonstrated reduced SVC density compared to non-carriers. The role of ApoE in the blood–brain barrier function has led to the hypothesis that it might impact this function either directly or indirectly by acting as a signaling molecule [10]. The SVC forms the inner blood–retina barrier and is suggested to reflect cerebral microcirculation [32]; given the close similarity between the blood–retina barrier and blood–brain barrier, the ε4 allele of ApoE might be linked to more permeable blood–retina barriers, potentially explaining the findings related to retinal microvasculature.

Elahi FM [37] reported reduced capillary density in clinically unimpaired APOE ε4 gene carriers based on SD-OCT. Similarly, we found microvascular densities were lower in APOE ε4 carriers compared to non-carriers in NCI, though the results are not significant. Device difference and sample size may account for inconsistency, and standardized OCTA studies with large sample sizes are required to validate the results. In the CI group, we found microvascular densities were significantly reduced in APOE ε4 carriers when compared to non-carriers. It is suggested that AD patients have the greatest degree of microvascular damage in the DVC [38,39] because the microvessels are thinner and have a smaller cross-section, which makes them sensitive to disease progression [40]. Our findings suggest that the APOE ε4 genotype is associated with retinal microvasculature changes dependent on the degree and level of cognitive function. Our study suggests that in APOE ε4 carriers, the pathological mechanism may be more selective and affect the SVC from the asymptomatic stage, suggesting that the superficial vessels of the retina may be sensitive to the earliest changes associated with AD pathology in older adults as previously reported [41].

Studying retinal microvascular changes in at-risk individuals can provide insight into how vascular contributions to AD can be studied. Interventions for AD may be more effective if they target biomarkers that reveal the earliest detectable abnormalities in the asymptomatic or preclinical stage rather than the clinical disease that is evident. This shift in perspective led to the development of biomarker-driven criteria for AD research based on amyloid, tau, and neurodegeneration (A/T/N) [42,43]. Research into how microvascular impairment can contribute to cognitive impairment and AD remains important and active. Modeling disease progression and clinical manifestation using in vivo imaging modalities such as OCTA may help detect high-risk individuals at an early stage. The retinal microvasculature imaged and measured with the OCTA could reflect on microvascular impairment that occurred in AD pathology, which may enable the assessment of purported treatments.

Our study has several limitations. The cross-sectional design of the study limits the interpretation of the results for cause and effect. The results were from a Chinese cohort, which limits the generalizability of the data to other races. Furthermore, individuals with ocular and neurological disorders that could affect our data were excluded, which might introduce selection bias.

## 5. Conclusions

In conclusion, we showed that older adults with CI had reduced SVC density compared to NCI. We also showed that APOE ε4 carriers showed reduced SVC density compared to APOE ε4 non-carriers. Importantly, in the CI group, APOE ε4 carriers showed reduced SVC and DVC densities compared to non-carriers. The microvascular changes observed in the sub-analysis of APOE genotype and cognitive status suggest that reduced retinal microvascular densities may be a subtle indicator in the AD continuum. Longitudinal studies of retinal and amyloid/tau pathology in the APOE ε4 cohort will help decipher the causality of vascular changes.

## Figures and Tables

**Table 1:** Demographics and clinical information of APOE ε4 non-carriers and ε4 carriers in all participants.

	All	APOE ε4 Non-Carriers	APOE ε4 Carriers	*p*-Value
*n*	163	128	35	
Age, years	59 (54–65)	59 (54–65)	58 (54–65)	0.810
Female, *n* (%)	102 (62.58%)	82 (64.06%)	20 (57.14%)	0.581
Education, years	9 (6–12)	9 (6–12)	9 (6–15)	0.395
Hypertension, *n* (%)	43 (26.71%)	35 (27.78%)	8 (22.86%)	0.714
Diabetes, *n* (%)	18 (11.18%)	16 (12.70%)	2 (5.71%)	0.366
Hyperlipidemia, *n* (%)	59 (36.65%)	50 (39.68%)	9 (25.71%)	0.187
Smoking, *n* (%)	13 (8.07%)	9 (7.14%)	4 (11.43%)	0.482
Drinkers, *n* (%)	14 (8.70%)	12 (9.52%)	2 (5.71%)	0.736
MMSE	29 (26–30)	29 (26–30)	29 (27–30)	0.281
MoCA	23 (20–26)	23 (20–26)	23 (21–27)	0.724
VA, LogMAR	0.2 (0.1–0.2)	0.2 (0.1–0.2)	0.1 (0.1–0.2)	0.304
OCTA parameters				
Eyes, *n*	311	242	69	
SVC, %	39.23 ± 5.42	39.61 ± 5.32	37.91 ± 5.58	0.021 *
DVC, %	50.22 ± 4.12	50.39 ± 3.92	49.64 ± 4.75	0.182

APOE: Apolipoprotein E, VA: visual acuity (VA), LogMAR: Logarithm of the Minimum Angle of Resolution, MMSE: mini-mental state examination, MoCA: Montreal Cognitive Assessment, SVC: superficial vascular plexus, and DVC: deep vascular plexus, *: *p* value < 0.05.

**Table 2:** Multivariate analysis of examining retinal vascular density differences of CI (143 eyes) vs. NCI (168 eyes) and APOE ε4 carriers (69 eyes) vs. non-carriers (242 eyes) in all participants.

	CI	NCI	*p*-Value	ε4 Carriers	ε4 Non-Carriers	*p*-Value
SVC, %	38.42 ± 5.32	39.91 ± 5.42	0.006 *	37.91 ± 5.58	39.61 ± 5.32	0.023 ^†^
DVC, %	49.92 ± 3.97	50.49 ± 4.25	0.231	49.64 ± 4.75	50.39 ± 3.92	0.221

NCI: non-cognitively impaired; CI: cognitively impaired; SVC: superficial vascular complex; DVC: deep vascular complex. * *p*-value was adjusted for age, gender, education, visual acuity, vascular risk factors (hypertension, diabetes mellitus, hyperlipidemia, and smoking), and intereye dependencies. ^†^ *p*-value was adjusted for age, gender, education, vascular risk factors (hypertension, diabetes mellitus, hyperlipidemia, and smoking), and intereye dependencies.

**Table 3:** Comparison of OCTA parameters between APOE ε4 carriers (69 eyes) vs. non-carriers (242 eyes) in subgroups of CI or NCI.

		ε4 Carriers	ε4 Non-Carriers	*p*-Value
NCI	SVC, %	39.05 ± 5.84	40.18 ± 5.28	0.796
	DVC, %	50.39 ± 4.84	50.51 ± 4.07	0.780
CI	SVC, %	36.42 ± 4.92	38.96 ± 5.32	0.006 *
	DVC, %	48.66 ± 4.53	50.25 ± 3.76	0.048 *

NCI: non-cognitively impaired; CI: cognitively impaired; SVC: superficial vascular complex; DVC: deep vascular complex. *p*-values were adjusted for age, gender, education, vascular risk factors (hypertension, diabetes mellitus, hyperlipidemia, and smoking), and intereye dependencies; *: *p* value < 0.05.

**Table 4:** Multivariate linear analysis of examining the relative contribution of APOE genotype and cognition to retinal microvascular density.

Variables	SVC			DVC		
-	B	SE	*p*-Value	B	SE	*p*-Value
APOE ε4 carriers	−1.638	2.588	0.024 *	−0.772	0.579	0.183
CI	−1.397	0.606	0.021 *	−0.674	0.485	0.166

APOE: Apolipoprotein E; CI: cognitively impaired; SVC: superficial vascular complex; DVC: deep vascular complex; B: partial regression coefficients; SE: standard error; *: *p* value < 0.05.

## Data Availability

The data that support the findings of this study are available on request from the corresponding author.

## Supplementary Materials

The following supporting information can be downloaded at: https://www.mdpi.com/article/10.3390/jcm12175649/s1. Figure S1: The results of interaction analysis of APOE genotype and cognition on SVC; Table S1: Demographics and clinical information of NCI and CI in all participants; Table S2: Permutation analysis of demographics and clinical information of APOE ε4 non-carriers vs. ε4 carriers in participants; Table S3: Permutation analysis of comparison OCTA parameters between APOE ε4 carriers vs. non-carriers in subgroup of CI or NCI; Table S4: cut-offs of CI.
