# Supplementary material for: APOE ε4 Gene Carriers Demonstrate Reduced Retinal Capillary Densities in Asymptomatic Older Adults
Source: J Clin Med. 2023 Aug 30;12(17):5649. doi: 10.3390/jcm12175649 (PMC10488535; doi:10.3390/jcm12175649)
Supplement: Supplementary file 1 [file jcm-12-05649-s001.zip › jcm-2511543-supplementary.docx]

**Supplementary material**

**Figure legends**

**Figure S1. The results of interaction analysis of** **APOE genotype and cognition on SVC.**

**
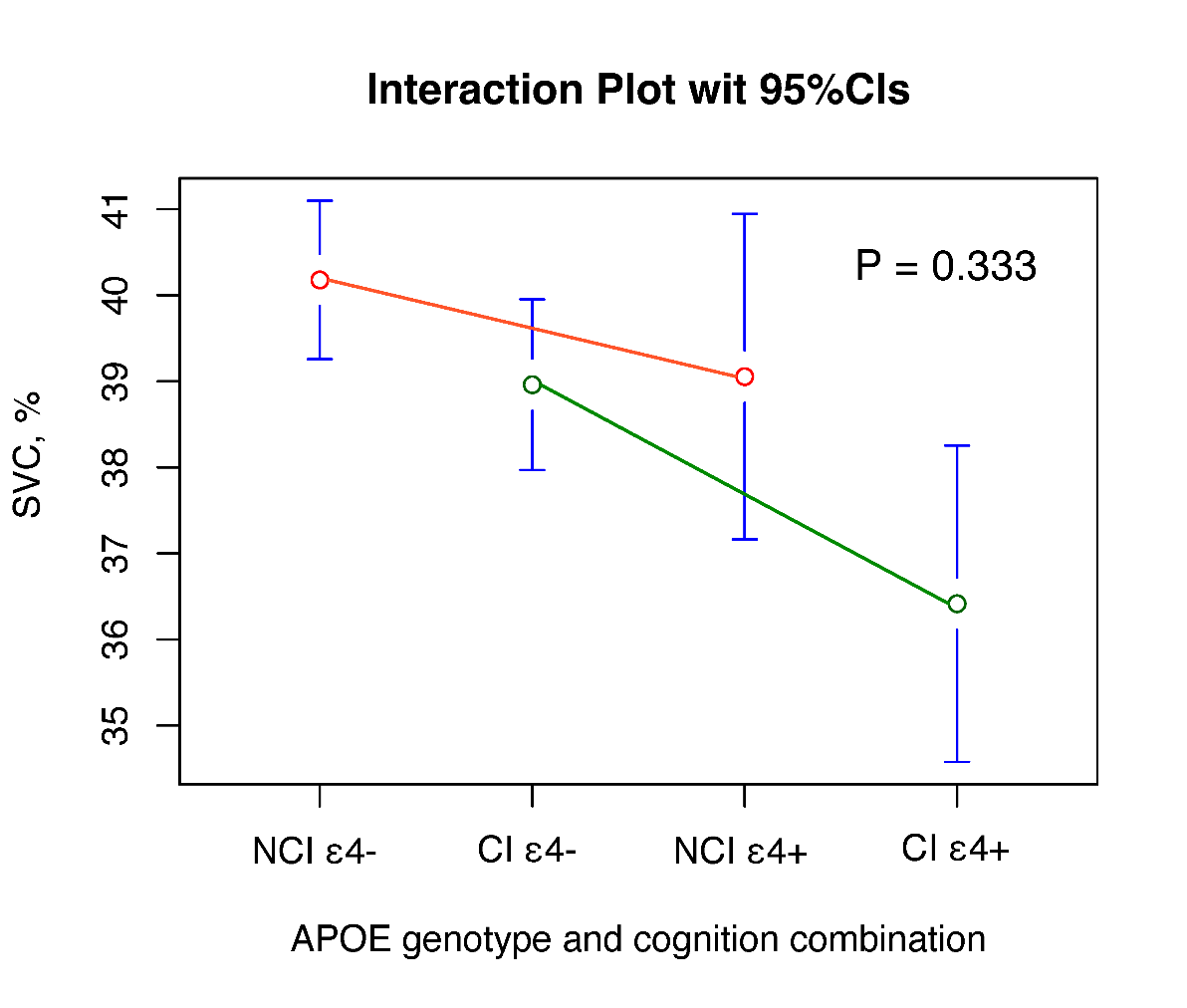
**

Table S1. Demographics and clinical information of NCI and CI in all participants.

|  | ALL | NCI | CI | P value |
| --- | --- | --- | --- | --- |
| n | 163 | 89 | 74 |  |
| Age, years | 59 (54 - 65) | 58(54 - 64) | 60(55 - 65) | 0.416 |
| Female, n (%) | 102 (62.58%) | 50 (56.18%) | 52 (70.27%) | 0.091 |
| Education, years | 9(6 - 12) | 9(6 - 14) | 9(6 - 12) | 0.661 |
| Hypertension, n (%) | 43 (26.71%) | 27 (31.03%) | 16 (21.62%) | 0.243 |
| Diabetes, n (%) | 18 (11.18%) | 7 (8.05%) | 11 (14.68%) | 0.213 |
| Hyperlipidemia, n(%) | 59 (36.65%) | 35 (40.23%) | 24 (32.43%) | 0.390 |
| Smoking, n (%) | 13 (8.07%) | 9 (10.34%) | 4 (5.41%) | 0.385 |
| Drinkers, n (%) | 14 (8.70%) | 10 (11.49%) | 4 (5.41%) | 0.261 |
| APOE ε4 carriers, n(%) | 35 (21.47%) | 20 (22.47%) | 15 (20.47%) | 0.881 |
| VA, LogMAR | 0.2(0.1 - 0.2) | 0.2(0.1 - 0.3) | 0.2(0.1 - 0.2) | 0.361 |
| MMSE | 29(26 - 30) | 30(28 - 30) | 28(25 - 29) | <0.001 |
| MoCA | 23(20 - 26) | 26(22 - 28) | 21(19 - 23) | <0.001 |
| OCTA parameters |  |  |  |  |
| SVC, % | 39.23±5.42 | 39.91±5.42 | 38.42±5.32 | 0.015* |
| DVC, % | 50.22±4.12 | 50.49±4.25 | 49.92±3.97 | 0.229 |
| APOE: Apolipoprotein E, VA: visual acuity (VA), MMSE: Mini-mental state examination, MoCA: Montreal Cognitive Assessment, SVC: superficial vascular plexus, DVC: deep vascular plexus, *: *p* value < 0.05. | | | | |

**Permutation test**

As for permutation test for t-test, Fisher-Pitman permutation test was used. The t0-statistics was calculated based on observational data; every new observed t-statistics was calculated after every random allocation of grouping label to observations. Then ranked all new observed t-statistics, and two tailed p value was the twice of proportion that new observed t-statistics greater than t0-statistics. All combination of allocation was considered (exact method); cion package in R was used to calculation.

As for permutation test for linear models, steps were similar; but approximate method was used because of computational complexity. Monte Carlo sampling method was used as approximate method; lmPerm package in R was used.

**Permutation results**

The differences for SVC density between APOE ε4 non-carriers vs ε4 carriers in permutation tests remained significant (p = 0.021, Supplementary Table 2). Permutation tests showed the same results as original tests in subgroup analysis, that in the NCI group, no significant differences (P > 0.05) were seen in the microvascular densities between APOE ε4 carriers and non-carriers; in the CI group, APOE ε4 carriers showed reduced SVC (P = 0.005) and DVC (P = 0.027) densities compared to non-carriers (Supplementary Table 3).

Table S2. Permutation analysis of demographics and clinical information of APOE ε4 non-carriers vs ε4 carriers in participants.

|  | All | ε4 non-carriers | ε4 carriers | P value |
| --- | --- | --- | --- | --- |
| n | 163 | 128 | 35 |  |
| Age, years | 59 (54 - 65) | 59(54 - 65) | 58(54 - 65) | 1 |
| Female, n (%) | 102 (62.58%) | 82 (64.06%) | 20 (57.14%) | 0.258 |
| Education, years | 9(6 - 12) | 9(6 - 12) | 9(6 - 15) | 0.231 |
| Hypertension, n (%) | 43 (26.71%) | 35 (27.78%) | 8 (22.86%) | 0.538 |
| Diabetes, n (%) | 18 (11.18%) | 16 (12.70%) | 2 (5.71%) | 0.131 |
| Hyperlipidemia, n (%) | 59 (36.65%) | 50 (39.68%) | 9 (25.71%) | 0.048 |
| Smoking, n (%) | 13 (8.07%) | 9 (7.14%) | 4 (11.43%) | 0.315 |
| Drinkers, n (%) | 14 (8.70%) | 12 (9.52%) | 2 (5.71%) | 0.352 |
| VA, LogMAR | 0.2(0.1 - 0.2) | 0.2(0.1 - 0.2) | 0.1 (0.1 - 0.2) | 0.076 |
| MMSE | 29(26 - 30) | 29(26 - 30) | 29(27 - 30) | 0.697 |
| MoCA | 23(20 - 26) | 23(20 - 26) | 23(21 - 27) | 0.667 |
| OCTA parameters |  |  |  |  |
| SVC, % | 39.23±5.42 | 39.61±5.32 | 37.91±5.58 | 0.021* |
| DVC, % | 50.22±4.12 | 50.39±3.92 | 49.64±4.75 | 0.187 |

APOE: Apolipoprotein E, VA: visual acuity (VA), MMSE: Mini-mental state examination, MoCA: Montreal Cognitive Assessment, SVC: superficial vascular plexus, DVC: deep vascular plexus, *: *p* value < 0.05.

Table S3. Permutation analysis of comparison OCTA parameters between APOE ε4 carriers vs non-carriers in subgroup of CI or NCI.

|  |  | ε4 carriers | ε4 non-carriers | P value |
| --- | --- | --- | --- | --- |
| NCI | SVC, % | 39.05±5.84 | 40.18±5.28 | 0.482 |
|  | DVC, % | 50.39±4.84 | 50.51±4.07 | 0.510 |
| CI | SVC, % | 36.42±4.92 | 38.96±5.32 | 0.005* |
|  | DVC, % | 48.66±4.53 | 50.25±3.76 | 0.027* |

NCI: non-cognitively impaired; CI: cognitively impaired; SVC: superficial vascular complex; DVC: deep vascular complex; P values were adjusted for age, gender, education, vascular risk factors (hypertension, diabetes mellitus, hyperlipidemia, smoking), and intereye dependencies; *: *p* value < 0.05.

Table S4. cut-offs of CI.

| Cognitive test | Education (years) | Borderlines of CI (scores) |
| --- | --- | --- |
| MoCA | 0 | ≤13 |
|  | 1-6 | ≤19 |
|  | 7-12 | ≤24 |
|  | ＞12 | ≤25 |
